# RSK1 and RSK2 serine/threonine kinases regulate different transcription programs in cancer

**DOI:** 10.3389/fcell.2022.1015665

**Published:** 2023-01-04

**Authors:** Won Seok Yang, Maisel J. Caliva, Vedbar S. Khadka, Maarit Tiirikainen, Michelle L. Matter, Youping Deng, Joe W. Ramos

**Affiliations:** ^1^ Cancer Biology Program, University of Hawaii Cancer Center, University of Hawaii at Mānoa, Honolulu, HI, United States; ^2^ Department of Quantitative Health Sciences, John A. Burns School of Medicine, University of Hawaii at Manoa, Honolulu, HI, United States

**Keywords:** RSK, transcription, microarray, cell cycle, immune response, kinase, adhesion

## Abstract

The 90 kDa ribosomal S6 kinases (RSKs) are serine threonine kinases comprising four isoforms. The isoforms can have overlapping functions in regulation of migration, invasion, proliferation, survival, and transcription in various cancer types. However, isoform specific differences in RSK1 versus RSK2 functions in gene regulation are not yet defined. Here, we delineate ribosomal S6 kinases isoform-specific transcriptional gene regulation by comparing transcription programs in RSK1 and RSK2 knockout cells using microarray analysis. Microarray analysis revealed significantly different mRNA expression patterns between RSK1 knockout and RSK2 knockout cell lines. Importantly some of these functions have not been previously recognized. Our analysis revealed RSK1 has specific roles in cell adhesion, cell cycle regulation and DNA replication and repair pathways, while RSK2 has specific roles in the immune response and interferon signaling pathways. We further validated that the identified gene sets significantly correlated with mRNA datasets from cancer patients. We examined the functional significance of the identified transcriptional programs using cell assays. In alignment with the microarray analysis, we found that RSK1 modulates the mRNA and protein expression of Fibronectin1, affecting cell adhesion and CDK2, affecting S-phase arrest in the cell cycle, and impairing DNA replication and repair. Under similar conditions, RSK2 showed increased ISG15 transcriptional expression, affecting the immune response pathway and cytokine expression. Collectively, our findings revealed the occurrence of RSK1 and RSK2 specific transcriptional regulation, defining separate functions of these closely related isoforms.

## Introduction

Transcription factors regulate gene expression by modulating the synthesis of messenger RNA thereby creating cell-specific gene expression patterns. They are key regulators of proliferation, differentiation, survival, motility, and apoptosis. Dysregulation of transcription may lead to abnormal gene expression that promotes tumor initiation and progression (Lee and Young, 2013; Bradner et al., 2017). Extracellular signals, including growth factors, hormones, cytokines, and environmental stresses, elicit changes in gene expression to initiate physiological responses (Whitmarsh, 2007). The most common ways in which gene expression is controlled is by phosphorylation and de-phosphorylation of transcription regulators, which are mediated by specific protein kinases and phosphatases (Hill and Treisman, 1995; Whitmarsh and Davis, 2000).

Mitogen-activated protein kinase (MAPK) signaling pathways alter the activity of transcription regulators by controlling their expression, stability, localization and binding affinity to target DNA or transcriptional complexes in cells (Hazzalin and Mahadevan, 2002; Yang et al., 2003). The 90 kDa ribosomal S6 kinases (RSK) are downstream effectors of the MAPK pathway and are directly activated by extracellular signal-regulated kinases (ERK) 1 and 2. The RSK family of proteins consists of four isoforms (RSK1-4) that share 73%–80% amino acid identity with the most significant variations in their N- and C-terminal sequences (Anjum and Blenis, 2008; Romeo et al., 2012). RSKs consist of two kinase domains connected by a linker region. The activation of RSKs requires phosphorylation by both ERK and phosphoinositide-dependent kinase 1 (PDK1), followed by auto-phosphorylation of the N-terminal kinase domain (NTKD) by the C-terminal kinase domain (CTKD). Upon activation the RSKs regulate downstream targets through phosphorylation by the NTKD (Sulzmaier and Ramos, 2013). mRNA expression of RSKs1-4 is detectable in all human tissues; RSK4 has the lowest expression and demonstrates tissue-specific variation in expression levels suggesting isoform-specific function as well as tissue-specific roles (Kohn et al., 2003; Dummler et al., 2005; Wu et al., 2009).

The RSK family contributes to diverse cellular processes including cell growth, survival, and motility. RSK1 and 2 promote tumor growth, whereas RSKs 3 and 4 have tumor-suppressive functions (Doehn et al., 2009; Romeo and Roux, 2011). For example, in melanomas, RSK1 promotes motility by phosphorylating p27 Kip1 to induce cytoplasmic localization and RhoA inhibition (Larrea et al., 2009). RSK1 inhibits pro-apoptotic activity by phosphorylating Bad, a member of the Bcl2 family (Shimamura et al., 2000). RSK2 suppresses integrin activation by phosphorylating Filamin A and regulates RhoA GTPases to promote cell motility (Gawecka et al., 2012; Shi et al., 2018). Moreover shRNA-mediated RSK2 knockdown in glioblastoma results in decreased cell motility and invasion (Sulzmaier et al., 2016). RSK 3 induces G1 phase cell cycle arrest and apoptosis when overexpressed in ovarian and breast cancer cells (Bignone et al., 2007). Lastly, RSK4 negatively regulates breast cancer cell invasion through its regulation of Claudin-2 and CXCR expression (Thakur et al., 2008).

RSK1 directly phosphorylates the serum response factors (SRF) (Rivera et al., 1993), c-Fos (Chen et al., 1993) and Nur77 (Davis et al., 1993; Wingate et al., 2006) to regulate transcription. Their phosphorylation promotes the degradation of IκBα/β, which leads to NF-κB activation (Ghoda et al., 1997; Schouten et al., 1997). RSK1 also associates with and phosphorylates estrogen receptor α (ERα) on Ser167 which increases ERα-mediated transcription (Joel et al., 1998; Yamnik and Holz, 2010). RSK2 promotes c-Fos transcription by phosphorylating both the Elk1/SRF complex and the cAMP response element-binding protein (CREB) (De Cesare et al., 1998; Bruning et al., 2000). RSK2 directly phosphorylates c-Fos, thereby increasing its stability for regulating cyclin D1 expression in the cell cycle (Chen et al., 1996; David et al., 2005). RSK1 can inhibit migration in lung cancer cells through phosphorylation of the actin-binding protein VASP, which leads to decreased metastatic behavior (Lara et al., 2011). A study using siRNA knockdown revealed that RSK2, but not RSK1, controls adhesion and rearrangement of the actin cytoskeleton to affect motility in head and neck squamous cell carcinoma (HNSCC) (Kang et al., 2010). These results indicate that further study is needed to understand the mechanisms of action of the different RSK isoforms in cancer biology.

We report that RSK1 and RSK2 have isoform-specific transcriptional programs in glioblastoma (GBM)-derived cell lines. Microarray analysis determined that RSK1 specifically regulated gene expression related to the cell adhesion, cell cycle and induced S phase arrest, whereas RSK2 modulated the immune response, resulting in induced cytokine secretion. Overall, our findings provide insight into the specific roles of RSK isoforms in regulating gene transcription and subsequent functional outcomes.

## Materials and methods

### Expression analysis of RSK1 and RSK2

GEPIA, a webserver that includes 9,736 tumors and 8,587 normal tissue samples from TCGA and GTEx projects, was used to generate RSK1 and RSK2 expression with the log-rank test in 4 different types of cancer (Tang et al., 2017).

### Cell culture

Human glioblastoma-derived U251 MG cells were a gift from Dr. Santosh Kesari (USCD School of Medicine, La Jolla, CA) and were validated by STR analysis. Cells were cultured in DMEM (Corning, MA) supplemented with 10% fetal bovine serum (Seradigm, Cat 1500–500) and 1% Antibiotic-Antimycotic (ThermoFisher Scientific, MA). Cells were incubated in a humidified chamber containing 5% CO_2_ at 37°C.

### Construction of clustered regularly interspaced short palindromic repeats plasmid

For oligo design, the first 200 bp within the coding region of each RSK was checked for homology within their isotypes as well as localization in a single exon. 20 bp target guide RNA (gRNA) sequences were designed using MIT’s CRISPR Design Tool (http://www.crispr.mit.edu). Oligonucleotide pairs of gRNA were annealed and subsequently inserted in lenti-CRISPR v2 (Sanjana et al., 2014) (a gift from Feng Zhang, Addgene #52961). The gRNA pairs for RSK1 were 5′-CAC​CGA​GCC​TTG​ACG​TGG​TGC​GTG​A-3` (fwd) and 5′-AAACTCAC GCA​CCA​CGT​CAA​GGC​TC-3` (rev); RSK2 were 5′-CAC​CGA​AGA​TGC​CGC​TGG​CGC​AGC-3` (fwd) and 5′-AAA​CGC​TGC​GCC​AGC​GGC​ATC​TTC-3` (rev).

### Generating clustered regularly interspaced short palindromic repeats/Cas9 knockout cell lines

For the generation of stable knockout cells, lentiviruses were generated in HEK 293 T cells using the lentivirus packing system. Packaging plasmid PMD2. G, PS PAX2 and lenti-CRISPR v2 were transfected with Lipofectamine 3,000 (Thermofisher). Following 48 h of lentivirus production, media was collected, cell debris removed by centrifugation and filtered through a 0.45 µm filter. Lentiviral media was supplemented with polybrene (10 μg/ml) and transduced to U251 cells in 6 well tissue culture dishes. After 72 h puromycin selection was initiated by exchanging the media with puromycin (4 μg/ml) containing media. Individual cells were separated by limited dilution into 96 well plates and resulting colonies were analyzed by Western blot to confirm specific gene knockout.

### Expression profiling by microarray chip assay

Cells were seeded in 6 well tissue culture plates and serum starved overnight prior to EGF (100 ng/ml) treatment for 5 h. Cells were washed with 1X cold PBS and collected for RNA isolation. Processing of RNA samples (three biological replicates from each knockout cell line) was performed at the Genomics and Bioinformatics Shared Resource, University of Hawaii Cancer Center, University of Hawaii. RNA sample integrity was checked on an Agilent 2,100 Bioanalyzer using RNA Nano chip. Samples were prepared for microarray hybridization as described in the Thermo Fisher Scientific GeneChip Whole Transcript (WT) Expression manual. Double-stranded cDNA was generated from 100 ng of total RNA. cRNA was synthesized using the WT cDNA Synthesis and Amplification Kit (Thermo Fisher Scientific). cRNA was purified and reverse transcribed into single-stranded (ss) DNA. Subsequently a combination of uracil DNA glycosylase (UDG) and apurinic/apyrimidinic endonuclease 1 (APE 1) was used to fragment ssDNA, which was then labeled with biotin (WT Terminal Labeling Kit, Thermo Fisher Scientific). In a rotating chamber, 2.3 μg DNA were hybridized to the Clariom S Human Array for 16 h at 45°C. After washing and staining on an Affymetrix Fluidics Station FS450 using pre-formulated solutions (Hyb, Wash & Stain Kit, Thermo Fisher Scientific), hybridized arrays were scanned on the Affymetrix GeneChip Array Scanner 3000-7G. The expression intensity data was extracted from the scanned images and stored as CEL files. Generated CEL files were normalized using the SST-RMA-GENE-FULL algorithm in the Transcriptome Analysis Console (TAC) 4.0 software. Genes with fold change greater than 2 and (False Discovery Rate) FDR adjusted *p*-value less than 0.05 were considered differentially expressed. CEL files normalization using the SST-RMA-GENE-FULL algorithm and differential expression analysis [one-way analysis of variance (ANOVA)] were carried out using the Transcriptome Analysis Console (TAC) 4.0 software. Genes with fold change greater than 2 and (False Discovery Rate) FDR adjusted *p*-value less than 0.05 were considered differentially expressed.

### Pathway and gene ontology analysis

TAC 4.0 was used to visualize heatmap of differentially expressed genes (DEGs) with fold changes greater than 2. Furthermore, DEGs were subjected to Ingenuity Pathway Analysis (IPA; QIAGEN Inc., https://www.ingenuity.com) to gain insight into network discovery. DAVID software (Huang da et al., 2009a; Huang da et al., 2009b) (https://david.ncifcrf.gov/) was used with default settings to perform functional enrichment analysis of DEGs, and several significant gene ontology (GO) terms and pathways were identified at *p*-value <0.05. GO terms were classified by “Biological Process,” “Molecular Function” and “Cellular Component.”

### Cell adhesion assay

To measure cell adhesion, cells were harvested with a non-enzymatic cell dissociation buffer (Cellstripper, Cellgro, Mediatech) and incubated with Calcein-AM (8 µM) for 15 min at room temperature. Cells were subsequently plated into 96- well plates for 1 h followed by 3 times of PBS washing. Adhered cells were measured and quantified using fluorescent by ELISA plate reader (Perkin Elmer Envision, Waltham, MA, United States). HA-RSK1 was transfected into U251 RSK1 KO cells using X-tremeGENE HP (Sigma) transfection reagent. After 24 h, transfected cells were starved in 0.1% FBS media overnight followed by cell adhesion assay.

### Quantitative real-time polymerase chain reaction

Total RNA was extracted and purified using Trizol reagent (ThermoFisher Scientific, MA) according to manufacturer’s protocol. The cDNA was then reverse-transcribed from 2 μg of total RNA using ProtoScript II Reverse Transcriptase kit (NEB, MA). Quantitative real-time PCR was done with SYBR green PCR master mix (Applied Biosystem, CA). The C(t) values were normalized using GAPDH. Primers used were as follows:

TRIM69 : 5′-GGA​GCA​ATG​TCT​CTT​AGC​CAA​GG-3` (fwd) and 5′-TCTCTGGTTGCCAGCACCTTCA-3` (rev); ITGA2: 5′-GTG​GCT​TTC​CTG​AGA​ACC​GA-3` (fwd) and 5′-GATCAAGCCGAGGCTCATGT-3` (rev); TACC3: 5′-TCT​TGG​GAG​CAC​TGG​ACA​TTC​C-3` (fwd) and 5′-TCC​AGG​TCC​TTC​TGG​CTG​TAC​T-3` (rev); CDK2: 5′-TTC​CAC​CAG​CAT​GGC​AAC​GTC​T-3` (fwd) and 5′-AGC​TCC​GCG​TAT​TTG​CTT​TGG​G-3` (rev); BUB1B: 5′-GTGGAAGAGACTGCACAACAGC-3` (fwd) and 5′-TCA​GAC​GCT​TGC​TGA​TGG​CTC​T-3` (rev); COL6A3: 5′-GCA​GCA​GCA​AGC​AGT​CAT​TG-3` (fwd) and 5′-ATC​CTG​TCC​GAT​TTC​CAG​CC-3` (rev); SHC4: 5′-ACG​AGT​CGA​TCA​CGT​CCT​TG-3` (fwd) and 5′-AAG​GTG​GCA​TCT​TCA​GTC​GG-3` (rev); IL1B: 5′-CCA​CAG​ACC​TTC​CAG​GAG​AAT​G-3` (fwd) and 5′-GTGCAGTTCAGTGATCGTACAGG-3` (rev); ISG15 : 5′-TGC​GAC​GAA​CCT​CTG​AAC​AT-3` (fwd) and 5′-TCGAAGGTCAGCCAGAACAG-3` (rev); GAPDH: 5′-GTC​TCC​TCT​GAC​TTC​AAC​AGC​G-3` (fwd) and 5′-ACC​ACC​CTG​TTG​CTG​TAG​CCA​A-3` (rev).

### XTT assay

Cells were seeded in 96-well culture plates at a concentration of 5×10^3^ cells/well. Cell viability was assessed for Day 2, 3 and 4 using the XTT kit (Biotium) according to manufacturer’s protocol. Absorbance was measured by ELISA plate reader (Perkin Elmer Envision, Waltham, MA, United States) at 500 nm with a reference wavelength at 650 nm.

### Western blotting

Cells were seeded in 6 well tissue culture plates and starved with 0.1% FBS media overnight before being treated with EGF (100 ng/ml) at indicated times. Cells were then washed once with PBS and lysed with RIPA lysis buffer containing protease inhibitor cocktail (Roche). Protein samples were resolved by 10% SDS–PAGE after boiling for 5 min in 4X SDS sample buffer. Resolved proteins were transferred to a nitrocellulose membrane and subsequently analyzed by immunoblot using antibodies specific for p-p53 (S20), p-RSK (S380), p-YB1 (S102), YB1 (Cell Signaling Technology), RSK1 (Abcam), RSK2, p-ERK (Y204), ERK (Santa Cruz), and *ß* tubulin (Proteintech).

### Multiplex analysis of cytokines

We quantified 14 cytokine/chemokine/growth factor biomarkers simultaneously using a Discovery Assay^®^ called the Human High Sensitivity T cell Discovery Array 14-Plex (Eve Technologies Corp, Calgary, AB, Canada). The multiplex assay was performed at Eve Technologies by using the Bio-Plex™ 200 system (Bio-Rad Laboratories, Inc., Hercules, CA, United States), and a Milliplex Human High Sensitivity T cell panel (Millipore, St. Charles, MO, United States) according to their protocol. The 14-plex consisted of GM-CSF, IFNγ, IL-1β, IL-2, IL-4, IL-5, IL-6, IL-8, IL-10, IL-12 (p70), IL-13, IL-17A, IL-23, and TNFα. The assay sensitivities of these markers range from 0.11–3.25 pg/ml. Individual analyte values and other assay details are available on Eve Technologies’ website or in the Milliplex protocol.

### Tumor necrosis factor alpha enzyme-linked immunosorbent assay assay

Secreted TNF alpha was detected using the TNF alpha HUMAN ELISA Kit (Life technologies, Carlsbad, CA, United States) according to manufacturer’s protocol. Briefly, supernatant from EGF (100 ng/ml) stimulated NT, RSK1 KO and RSK2 KO cells were harvested and subsequently added to 96 well plates coated with TNF alpha. TNF alpha specific Biotin conjugation was labeled with Streptavidin-HRP for detecting the absorbance of the samples at 450 nm using ELISA plate reader (Perkin Elmer Envision).

### Cell cycle analysis

Cells were seeded in 100 mm dishes and starved overnight to synchronize the cells and arrest them in the G0/G1 phase of the cell cycle. Cells were treated with EGF (100 ng/ml) for 24 h followed by trypsinization and fixation with methanol. Fixed cells were washed with 1X PBS two times and treated with RNAse for 20 min at RT and then stained with Propidium Iodide in the dark before analysis. For cisplatin treated samples, starved cells were changed to 10% FBS media with or without 1 μM of cisplatin and incubated for 24 h. Cells were sorted by Accuri C6 Flow Cytometer (BD bioscience) from UHCC Microscopy and Imaging Core. Cell cycle distribution was analyzed with FlowJo software (Treestar, Ashland, OR). Both debris and doublets were removed from the analysis.

### Statistical analysis

The student’s two-tailed *t*-test was employed to determine the difference between control and treatment groups of cell-based assays (three independent experiments performed for analysis). The *p*-value less than 0.05 was considered statistically significant. Data are presented as mean ± SD.

## Results

### RSK1 and RSK2 demonstrate similar expression in diverse cancer types

**FIGURE 1:**
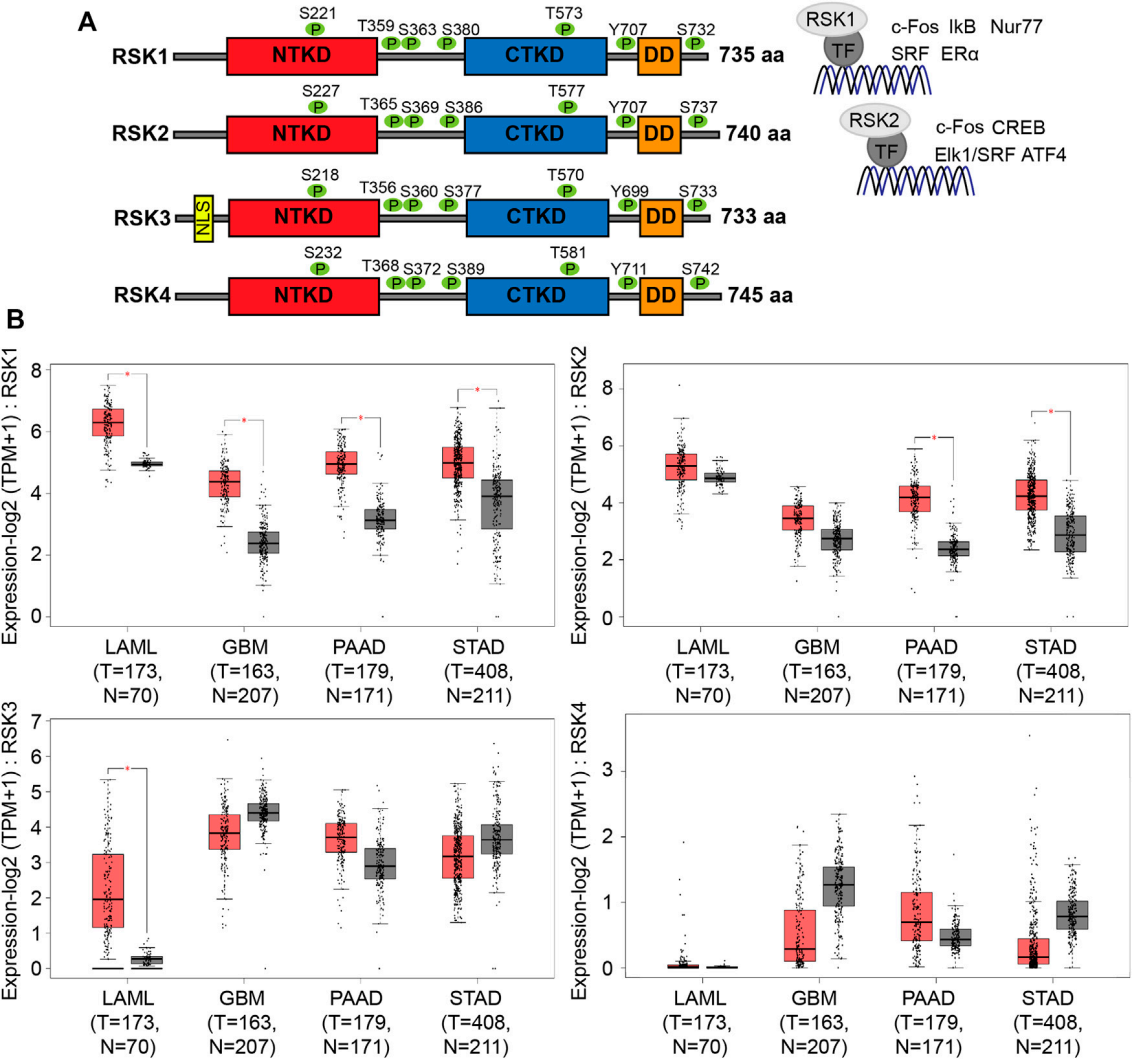
Structural and mRNA expression profiles of RSK isoforms in multiple tumors. **(A)** Schematic representation shows the functional domains of the N-terminal kinase domain (NTKD), C-terminal kinase domain (CTKD), and docking domain (DD). Activation of RSK is associated with highly conserved phosphorylation sites (each shown as green colored circles). Transcription factors regulated by RSK1 and RSK2 are shown on the right. **(B)** Box plots show tumor (red) and normal samples (gray) that revealed upregulation of RSK1 and RSK2 in acute myeloid leukemia (LAML), glioblastoma multiforme (GBM), pancreatic adenocarcinoma (PAAD), and stomach adenocarcinoma (STAD). The height of each bar represents the median expression of certain tumor or normal tissues (T = Tumor, N=Normal). Data were analyzed using the gene expression profiling interactive analysis 2 (GEPIA2) database. *, *p* < 0.05.

RSK isoforms have high homology (73%, 70% and 65% of protein sequence identity), primarily within the NTKD and CTKD. They have identical phosphorylation sites that are activated by ERK (RSK1-T573/RSK2-T577/RSK3-T570/RSK4-T581), leading to autophosphorylation (RSK1-S380/RSK2-S386/RSK3-S377/RSK4-S389) by the CTKD. Activation of the CTKD generates a docking site for PDK1, which then phosphorylates the NTKD (RSK1-S221/RSK2-S227/RSK3-S218/RSK4-S232), resulting in full activation of the RSKs. The activated RSK isoforms target many proteins including transcriptional factors. RSK1 can target the transcription factors c-Fos (Chen et al., 1993), IκB (Schouten et al., 1997), Nur77 (Wingate et al., 2006), SRF (Rivera et al., 1993), and ERα (Joel et al., 1998), whereas RSK2 can target c-Fos (David et al., 2005), CREB (De Cesare et al., 1998), Elk1/SRF (Bruning et al., 2000), and ATF4 (Yang et al., 2004) (Figure 1A). Although the specificity of each of the RSK isoforms for these transcription factors was not tested, these studies revealed the important role of RSK1 and 2 in regulating transcription. The expression of RSK isoforms mRNA in various tumors are shown (Figure 1B). To examine the expression levels of RSK1 and RSK2, tumor mRNA levels were analyzed from the gene expression profiling interactive analysis (GEPIA2) which computed TCGA data in the form of transcripts per million. Both RSK1 and RSK2 had high mRNA expression in acute myeloid leukemia (LAML), GBM, pancreatic adenocarcinoma (PAAD), and stomach adenocarcinoma (STAD) patient data. The expression of RSK3 and RSK4 did not show significant difference in indicated tumors except the RSK3 expression in LAML. RSK 3 and RSK4 levels are very low and difficult to detect in the cell lines we use (U251) so we did not further examine these. The resulting boxplots revealed that RSK1 and RSK2 expression levels are consistently high in many cancers.

### Microarray analysis of RSK1 and RSK2 null glioblastoma multiforme-derived cell lines

**FIGURE 2:**
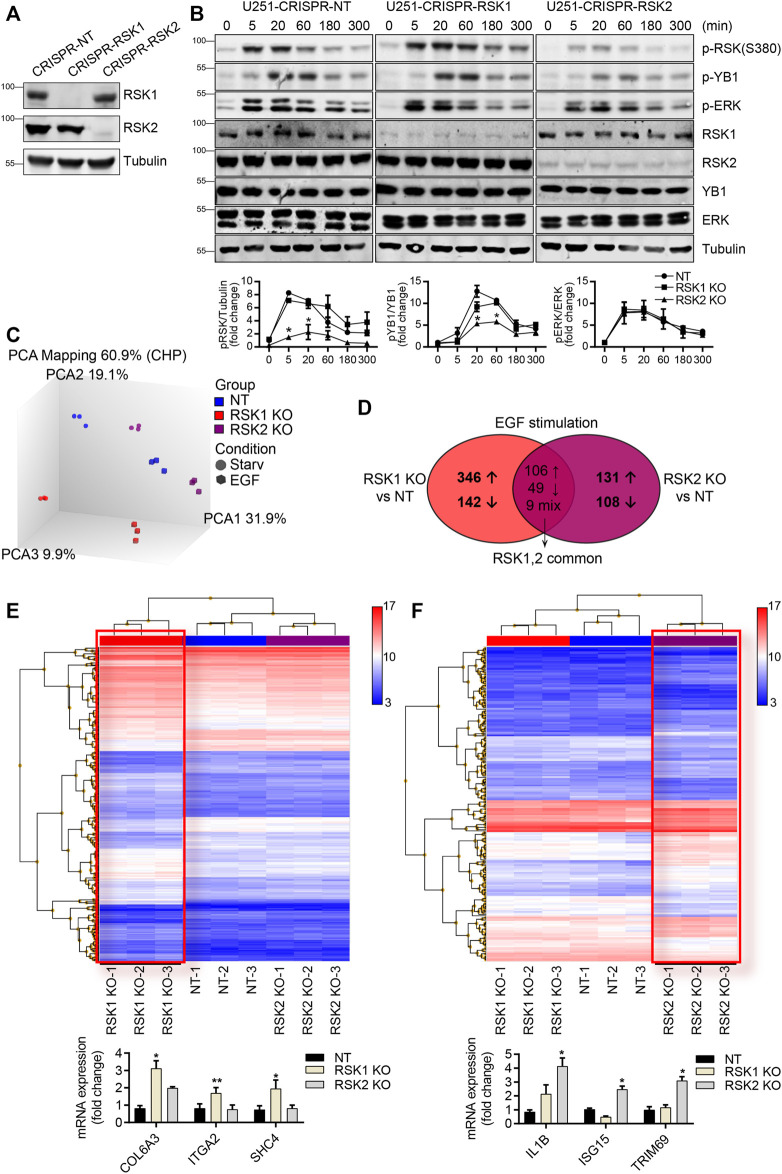
CRISPR/Cas9 mediated knockout of RSK1 and RSK2 for microarray analysis. **(A)** Stable knockout (KO) cell lines of RSK1 and RSK2 were confirmed by Western blotting. **(B)** Western blotting with indicated proteins after 100 ng/ml EGF treatment for 0, 5, 20, 60, 180, and 300 min. Each cell line was cultured in reduced serum (0.1% FBS) overnight. Tubulin was used as a loading control. Expression intensity of p-RSK, p-YB1 and p-ERK were quantified using ImageJ (bottom). **(C)** Principal component analysis (PCA) plot of overall mRNA expression data characterizing the entire transcriptional profile of the enrolled samples, generated using Transcriptome Analysis Console (TAC) software. **(D)** Venn diagram illustration of the numbers of differentially expressed genes (DEGs) specific to RSK1 KO and RSK2 KO, plus overlapping genes common to both RSK1 KO and RSK2 KO (fold change < −2 or >2; *p* < 0.05) in EGF stimulation condition. Arrows show up- and downregulated DEGs; mixed arrows represent opposite expression trends between the two isoforms. **(E)** Hierarchically clustered heat map illustrating DEGs between RSK1 KO, as highlighted in red box, and NT and RSK2 KO cell lines. mRNA expression of COL6A3, ITGA2 and SHC4 genes were used to validate microarray analysis (bottom). *, *p* < 0.05, **, *p* < 0.01. **(F)** Hierarchically clustered heat map illustrating DEGs between RSK2 KO, as highlighted in red box, and NT and RSK1 KO cell lines. mRNA expression of IL1B, ISG15 and TRIM69 genes were used to validate microarray analysis (bottom) *, *p* < 0.05. DEGs were from fold change >2.0, *p* < 0.05 and FDR <0.05, n = 3 per group.

RSK2 regulates drug resistance, cell motility, and invasion in many cancers including GBM (Sulzmaier and Ramos, 2013; Sulzmaier et al., 2016; Shi et al., 2018). However, specific gene regulation by RSK isoforms remains elusive. To determine if RSK1 and RSK2 have specific non-overlapping roles in gene expression, we used the clustered regularly interspaced short palindromic repeats (CRISPR)/Cas9 system to generate isoform-specific deletions in the GBM derived cell line U251 (Figure 2A). Cells were serum starved (0.1% FBS) overnight to minimize RSK activity, then stimulated with epidermal growth factor (EGF) (100 ng/ml) for various times. EGF activates ERK phosphorylation of both RSK1 and RSK2 as expected (Figure 2B). All three cell lines showed increased ERK activity after EGF stimulation, indicating that ERK is not affected by downstream CRISPR/Cas9 knockout of RSK1 and RSK2. In contrast, the phosphorylation of YB1, which is downstream of RSK, was inhibited in RSK2 knockout cells compared with control cells. Since decreased phosphorylation of YB1 was observed predominantly in RSK2-deleted cells, it follows that YB1 regulation depends predominantly on RSK2, but not RSK1 activity in these cells (Figure 2B).

To determine differences in mRNA programs driven by RSK1 versus RSK2, all three cell lines [control non-target (NT), RSK1 knockout (RSK1 KO), and RSK2 knockout (RSK2 KO)] were starved overnight to minimize RSK activity. The cells were then stimulated with EGF (100 ng/ml) for 5 h, followed by microarray analysis to compare differences in gene expression before and after EGF stimulation among the different cell types. Principal component analysis (PCA) of the microarray data revealed clusters within the biological replicates, with a marked separation between the 0.1% FBS and EGF-stimulated conditions for each cell line (Figure 2C). We then determined the differential gene expression among each group, using log2 fold change values of greater than 1 and *p* < 0.05. Each treated group (RSK1 KO and RSK2 KO) was compared separately with the control group (NT). Comparing RSK1 KO cells with NT cells under EGF stimulation conditions yielded a total of 652 detected differentially expressed genes (DEGs), whereas comparing RSK2 KO cells with NT cells under these same conditions yielded a total of 403 detected DEGs. The number of overlapping DEGs between RSK1 KO and RSK2 KO cells were also shown in the Venn diagram (Figure 2D). Of the 164 overlapping DEGs, 106 were upregulated, 49 were downregulated, and 9 were oppositely regulated between RSK1 KO and RSK2 KO. After excluding the overlapping DEGs, we were able to clarify 488 DEGs specific to RSK1 KO (346 upregulated and 142 downregulated), and 239 DEGs specific to RSK2 KO (131 upregulated and 108 downregulated). Transcriptome Analysis Console (TAC) software was used to overlay the up- and downregulated DEGs into hierarchical clustering graph from the comparison of triplicated NT, RSK1 KO and RSK2 KO cell lines (Figures 2E,F). Each cluster of RSK1 KO and RSK2 KO specific DEGs (highlighted in the red box) showed significantly differentiated expression levels according to the specific RSK isoform deletion. We also tested the mRNA expression of some DEGs from RSK1 KO (COL6A3, ITGA2 AND SHC4) and RSK2 KO cells (IL1B, ISG15 AND TRIM69) to confirm the results of the microarray. The validated DEGs were selected to support the isoform specific pathways which is shown below. In both PCA (Figure 2C) and hierarchical clustering graphs (Figures 2E,F), we observed significant separate clustering of each sample among NT, RSK1 KO and RSK2 KO cell lines.

### Pathway enrichment analysis of the differentially expressed genes

**FIGURE 3:**
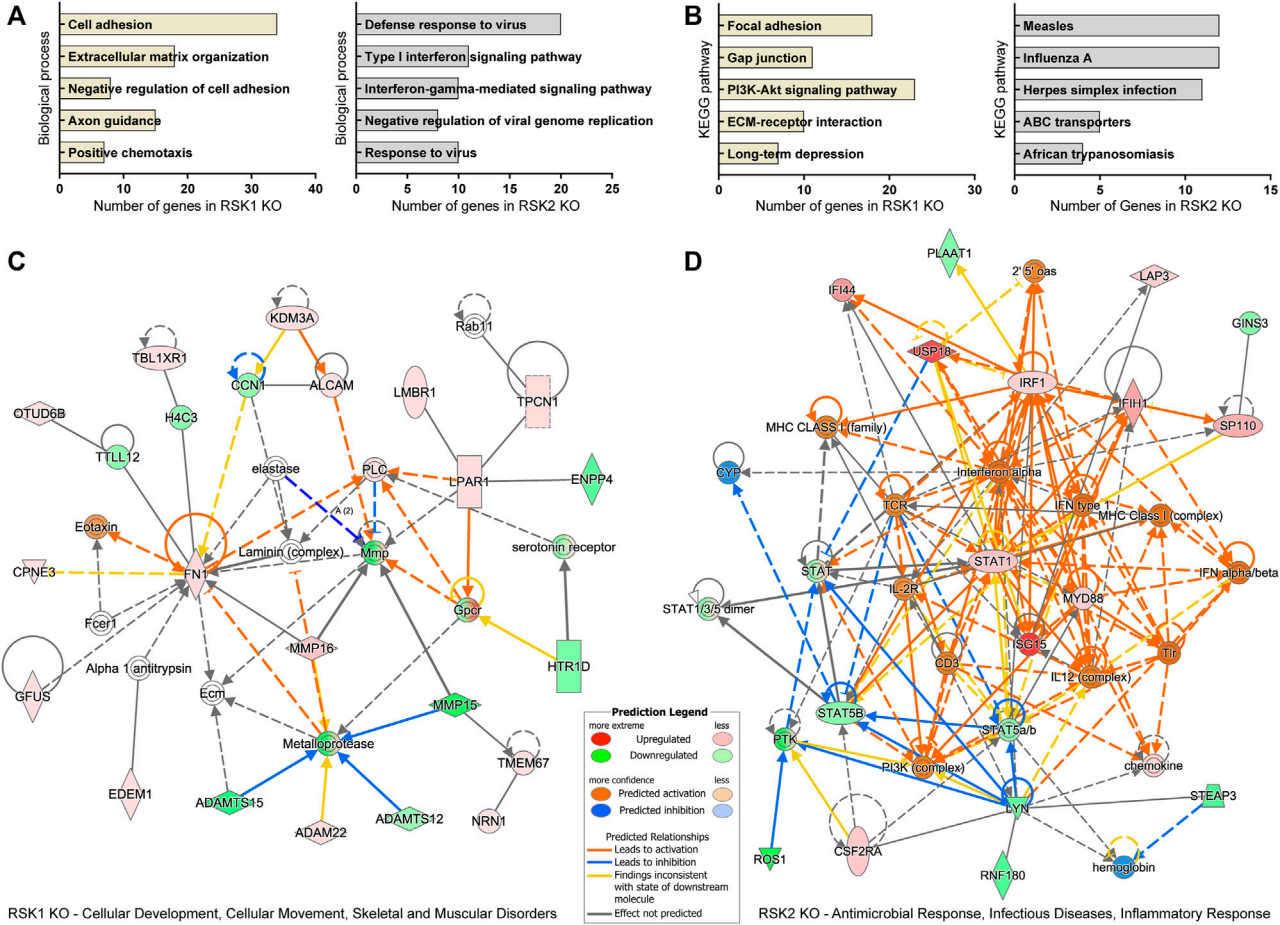
Gene Ontology (GO) analysis of biological processes, KEGG pathway and IPA network pathway analysis of DEGs specific to RSK1 KO and RSK2 KO. **(A)** Top five biological processes analyzed from RSK1 KO-specific DEGs (left) and RSK2 KO specific DEGs (right) in EGF stimulation. All collected biological processes were generated using the Database for Annotation, Visualization, and Integrated Discovery (DAVID). Bars represent the number of genes related to the indicated pathways (*p* < 0.05). **(B)** Top five KEGG pathways analyzed from RSK1 KO-specific DEGs (left) and RSK2 KO-specific DEGs (right) in EGF stimulation. **(C)** Gene network for ‘Cellular Development, Cellular Movement, Skeletal and Muscular Disorders’ from RSK1 KO-specific DEGs. **(D)** Gene network for ‘Antimicrobial Response, Infectious Diseases, Inflammatory Response) from RSK2 KO-specific DEGs. Gene networks based on IPA scores were identified.

Enrichment analysis of DEGs can give insight into cellular functions. We carried out detailed Gene Ontology (GO), KEGG (Kyoto Encyclopedia of Genes and Genomes) pathway analysis and IPA top network analysis of DEGs specific to RSK1 KO and RSK2 KO, under EGF stimulated condition. The DEGs were subjected to GO analysis based on biological processes using the Database for Annotation, Visualization, and Integrated Discovery (DAVID). The primary GO terms for enrichment of biological processes targeted by RSK1 KO-specific DEGs included the cell adhesion, extracellular matrix organization, negative regulation of cell adhesion, axon guidance and positive chemotaxis (Figure 3A, left). Biological processes targeted by RSK2 KO-specific DEGs included the defense response to virus, type I interferon signaling pathway, interferon-gamma mediated signaling pathway, negative regulation of viral genome replication and response to virus (Figure 3A, right). The KEGG pathway analysis of RSK1 KO and RSK2 KO specific DEGs also provided insight into the cellular pathways associated with these DEGs. Focal Adhesion and Gap junction were the top pathway enriched in RSK1 KO specific DEGs (Figure 3B, left). Measles and Influenza A were the top pathway enriched in RSK2 KO specific DEGs (Figure 3B, right). A network comparison analysis was conducted to identify interactions between DEGs in a given pathway and how they might work together at the molecular level. All DEG data from the TAC analysis were transferred into Ingenuity Pathway Analysis (IPA) software. Under EGF stimulated conditions, one of the top network related to RSK1 KO-specific DEGs was “Cellular Development, Cellular Movement, Skeletal and Muscular Disorders” (Figure 3C). And the top network related to RSK2 KO-specific DEGs was “Antimicrobial Response, Infectious Diseases and Inflammatory Response” (Figure 3D). Overall, these data indicate the role of RSK1 that regulates focal adhesion while RSK2 regulates immune response in GBM cell lines.

### RSK1 regulates cell adhesion whereas RSK2 regulates immune response

**FIGURE 4:**
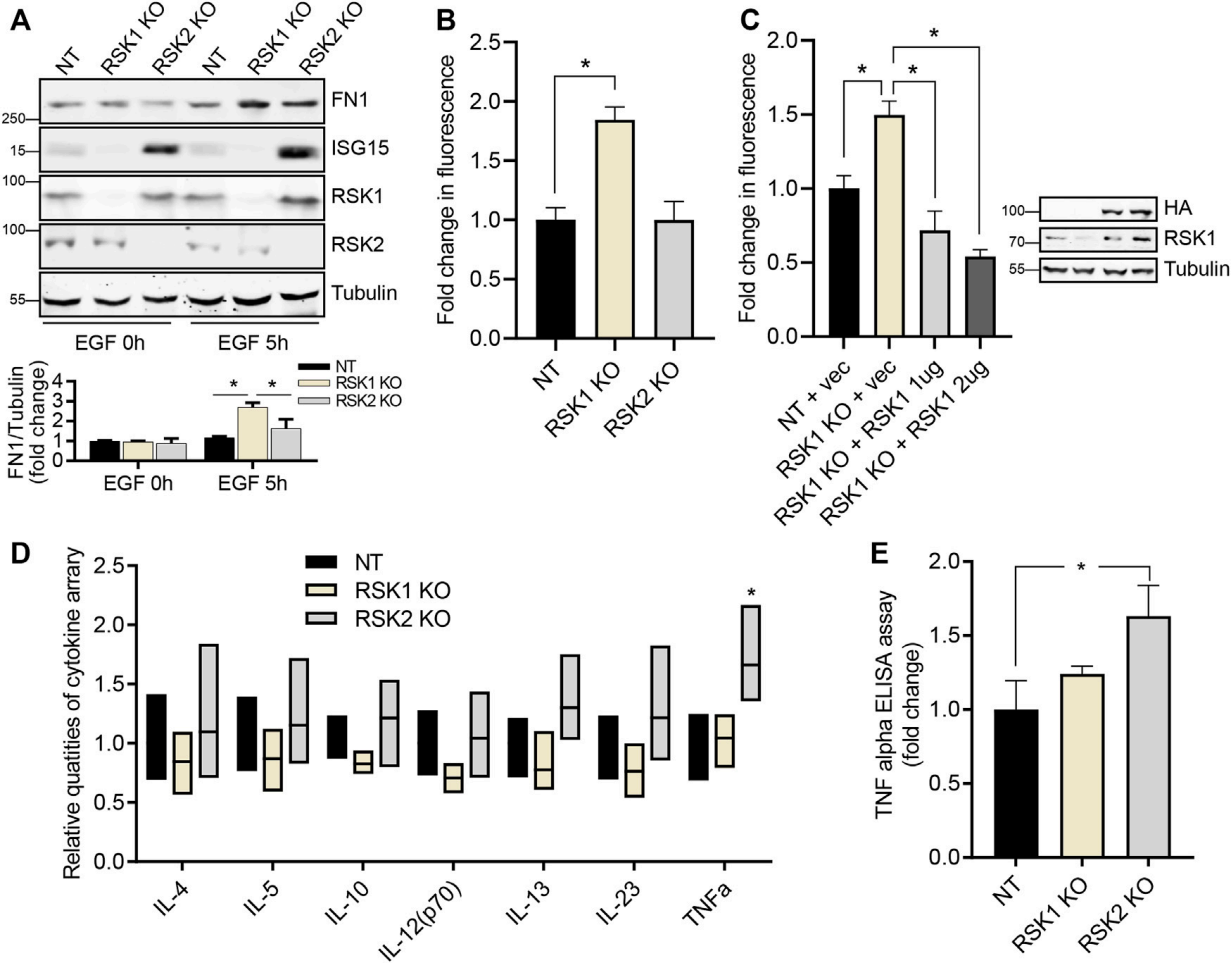
RSK1 regulates cell adhesion while RSK2 regulates immune response. **(A)** Representative western blot analysis of three cell lines in 0.1% FBS starved conditions, and after EGF stimulation. Tubulin was used as an internal control. Expression intensity of FN1 was quantified using ImageJ (bottom). **(B)** Cell adhesion assay shows the *in vitro* binding profile of NT, RSK1 KO and RSK2 KO cell lines. Calcein-AM staining of bound cells were measured and quantified. **(C)** Cell adhesion assay of restored RSK1 expression in RSK1 KO cell lines. Two different amounts of RSK1 was transiently transfected in RSK1 KO cell lines for the assay. The expression of RSK1 was confirmed by western blot analysis (right). **(D)** Bar graph showing the seven quantified cytokine/chemokine/growth factor biomarkers of three cell lines after EGF stimulation. Supernatant was collected and analyzed with a Human High Sensitivity T cell Discovery Array 14-Plex. **(E)** TNF alpha expression of three cell lines after EGF stimulation was quantified using an ELISA kit. All bar graphs represent average values of three experiments, with error bars indicating the standard deviation. *, *p* < 0.05.

To validate that pathway results from the microarray data were associated with related cellular functions in cell adhesion and immune response, we performed *in vitro* experiments. We first validated the protein expression level of DEGs shown in network comparison analysis (Figures 3C,D) through IPA. Fibronectin-1 (FN1) as an important protein in cell adhesion and ISG15 which was the most increased gene in network comparison analysis were tested. We found that the expression level of FN1 was increased after 5 h of EGF stimulation in RSK1 KO cell line and the expression level of ISG15 was also increased after EGF treatment in RSK2 KO cell line supporting the results of microarray analysis (Figure 4A). To confirm the pathway analysis results and induced expression of FN1, an extracellular matrix glycoprotein, which is known to important roles in cell adhesion and migration (Pankov and Yamada, 2002; To and Midwood, 2011), we tested cell adhesion activity of NT, RSK1 KO and RSK2 KO cells. After 5 h of EGF stimulation, we found that the cell adhesion activity of RSK1 KO cells were increased (1.8X fold) compared to NT and RSK2 KO cells supporting the results of microarray data (Figure 4B). We also tested that restoring RSK1 by exogenous expression led to decrease the cell adhesion in RSK1 KO cell line indicating direct regulation of cell adhesion in RSK1 (Figure 4C).

Next, we analyzed cytokines from cultured media to validate the enhanced immune response pathway seen in RSK2 KO cells under EGF-stimulated conditions. After EGF stimulation, fourteen cytokines/chemokines were analyzed from the three cell lines; of these, seven showed increased expression in the media. Tumor necrosis factor (TNF) alpha showed statistically significant expression in the RSK2 KO cells, while the other six cytokines showed a modest, but statistically insignificant increase in expression in these cells (Figure 4D). The TNF alpha expression level was re-confirmed using a TNF alpha enzyme-linked immunosorbent assay (ELISA) detection kit; this likewise showed increased expression in the RSK2 KO cells, indicating the presence of enhanced immune response pathways (Figure 4E). Collectively, these results strongly support that RSK1 mediates cell adhesion signaling pathways, while RSK2 mediates immune response signaling pathways.

### RSK1 regulates cell cycle-related pathways

**FIGURE 5:**
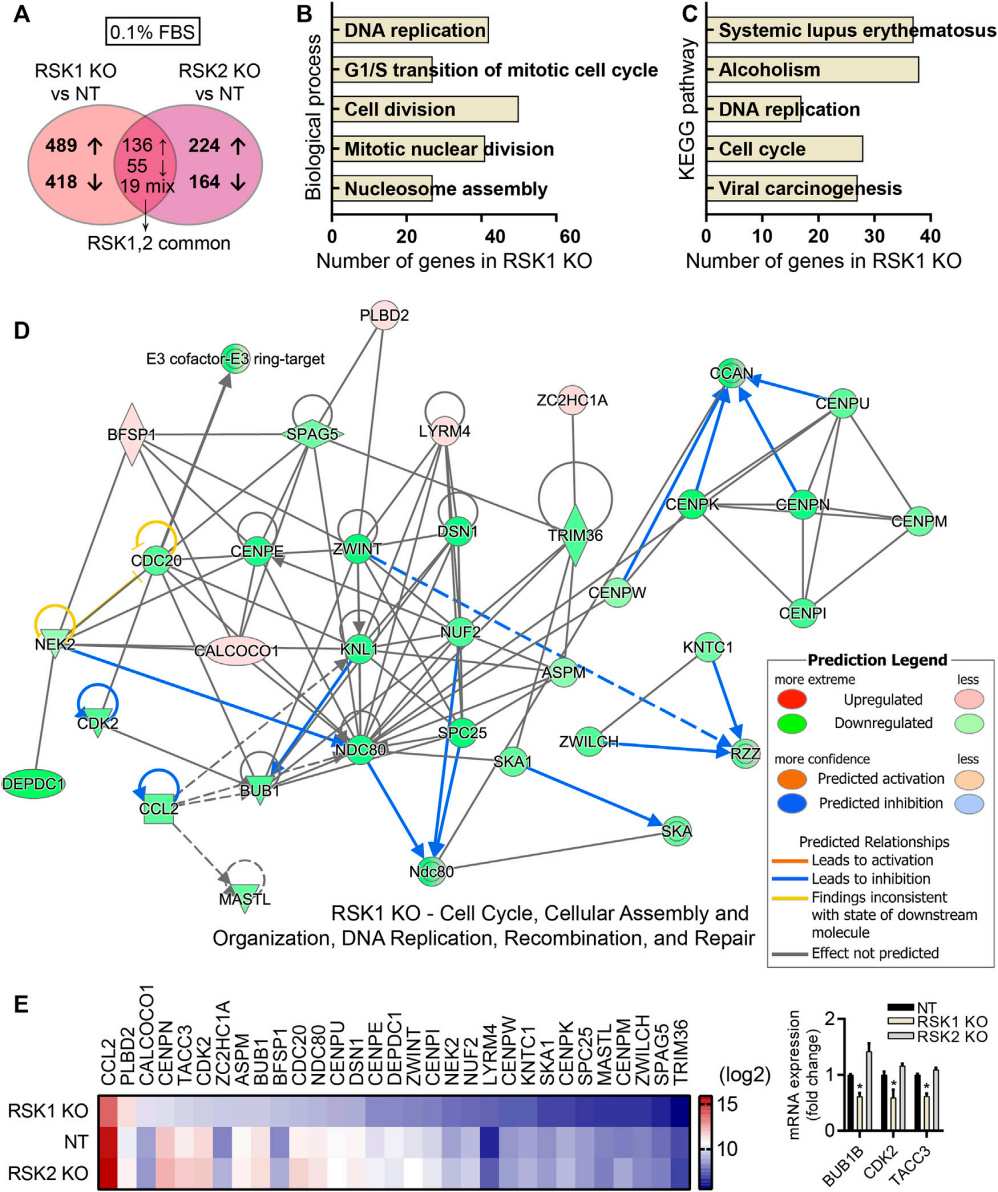
Pathway analysis of RSK1 KO DEGs in 0.1%FBS condition. **(A)** Venn diagram illustration of the numbers of DEGs specific to RSK1 KO and RSK2 KO, plus overlapping genes common to both RSK1 KO and RSK2 KO (fold change < -2 or >2; *p* < 0.05). **(B)** Top five biological processes analyzed from RSK1 KO-specific DEGs. **(C)** Top five KEGG pathways analyzed from RSK1 KO-specific DEGs. **(D)** Gene network for ‘Cell cycle, Cellular Assembly and Organization, DNA Replication, Recombination, and Repair’ from RSK1 KO-specific DEGs in 0.1% FBS. **(E)** Heat map analysis of thirty DEGs highlighted from top networks as shown in **(D)**. Increased (red) or decreased (blue) gene color intensity indicates a corresponding increased or decreased signal intensity, as annotated in the scale. mRNA expression of BUB1B, CDK2 and TACC3 genes were used to validate microarray analysis (right). *, *p* < 0.05.

The important roles of kinase activity independent mechanisms of many different types of kinases had been previously reported (Erazo et al., 2013; Yang et al., 2013; Malireddi et al., 2020). We therefore tested whether non-kinase activity of RSK1or RSK2 would affect the cellular pathways of RSK1 KO and RSK2 KO specific DEGs under 0.1% FBS (unstimulated) condition. Each group (RSK1 KO and RSK2 KO) was compared separately with the control group (NT) under 0.1% FBS conditions. After excluding the 210 overlapping DEGs, we were able to clarify 907 DEGs specific to RSK1 KO (489 upregulated and 418 downregulated), and 338 DEGs specific to RSK2 KO (224 upregulated and 164 downregulated). The number of DEGs between RSK1 KO and RSK2 KO cells were also shown in the Venn diagram (Figure 5A). The GO analysis of biological processes targeted by RSK1 KO-specific DEGs included the DNA replication, G1/S transition of mitotic cell cycle, Cell division, Mitotic nuclear division, and Nucleosome assembly (Figure 5B). The GO analysis of biological process targeted by RSK2 KO specific DEGs showed Cellular response to tumor necrosis factor, Positive regulation of peptidyl-Tyr-phosphorylation, Signal transduction, Response to drug and Cellular response to interlerkin-1 (data not shown). We then decided to focus on RSK1 KO specific DEGs since the pathway analysis pointed out that the cell cycle and DNA replication pathways are significantly involved in RSK1 KO cells. The KEGG pathway analysis of RSK1 KO specific DEGs also provided the cellular pathways of DNA replication and cell cycle which were one of the top pathways enriched in RSK1 KO specific DEGs (Figure 5C). In addition, a network comparison analysis was conducted into IPA software. Under 0.1% FBS condition, one of the top networks related to RSK1 KO-specific DEGs was “Cell Cycle, Cellular Assembly and Organization, DNA Replication, Recombination and Repair” (Figure 5D). The top network pathways related to the DEG expression panels were shown as a heat map to visualize their specific expression under certain conditions (Figure 5E, left). Most of the DEGs were downregulated compared to NT or RSK2 KO cells indicating significant reduced activity of DNA replication and cell cycle related pathways. We also tested the mRNA expression of some DEGs from RSK1 KO (BUB1B, CDK2, TACC3) to confirm the results of the microarray (Figure 5E, right). Together these results support the conclusion that under 0.1% FBS (unstimulated) conditions, DEGs specific to RSK1 KO were highly related to the cell cycle and DNA replication pathways.

**FIGURE 6:**
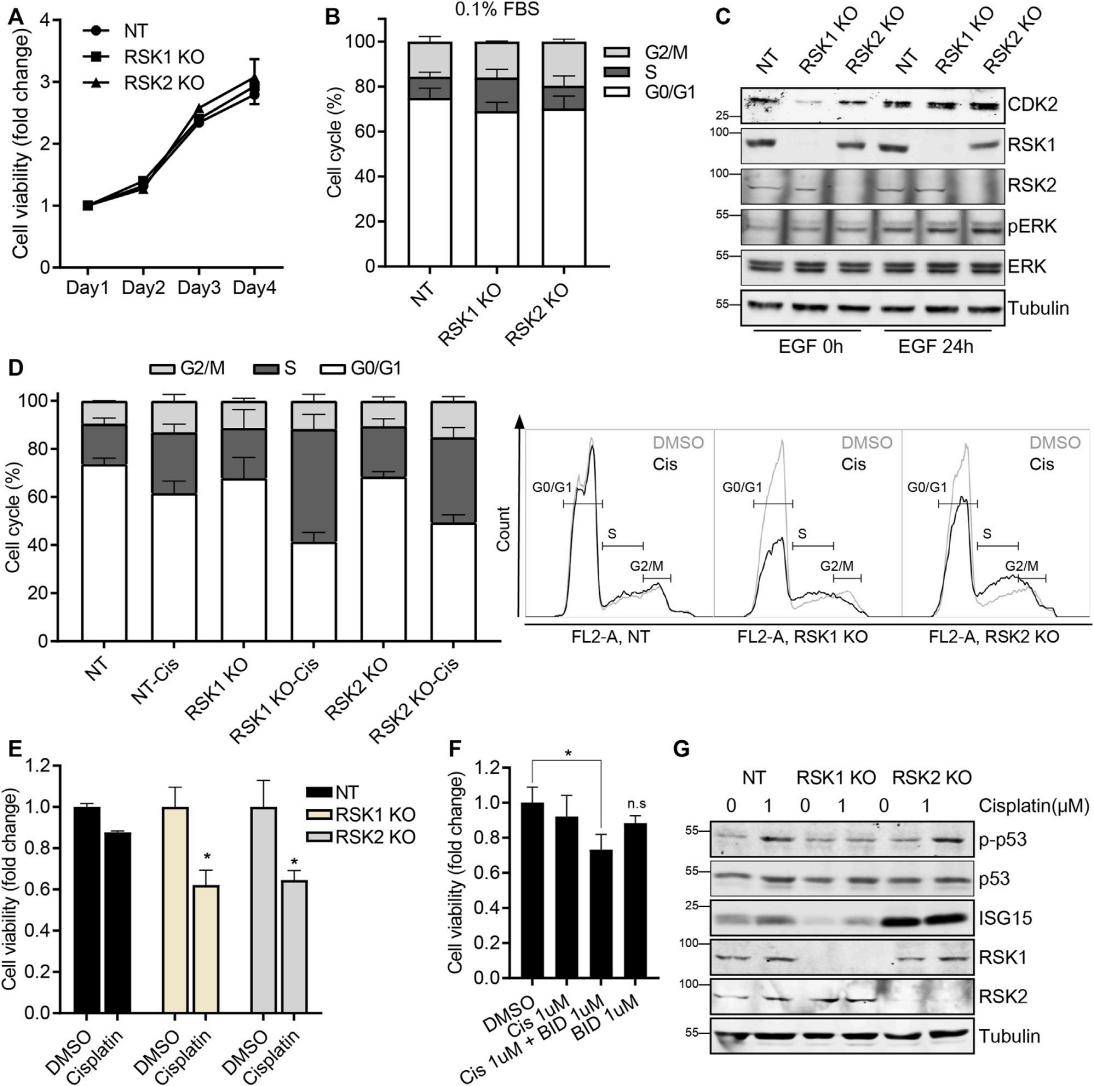
RSK1 regulates cell cycle and DNA repair pathways **(A)** XTT assay of U251 non-target (NT), RSK1 KO, and RSK2 KO cells. **(B)** Cell cycle analysis of three cell lines after starving in 0.1% FBS overnight. The bar graph shows the percentage of cells in each phase of the cell cycle (G0/G1, S, and G2). **(C)** Representative western blot analysis of three cell lines in 0.1% FBS starved conditions, and after EGF stimulation. pERK was used as a positive control for the EGF treatment, and tubulin was used as an internal control. **(D)** Cell cycle analysis of three cell lines after 24 h cisplatin (1 µM) treatment, and the resulting S-phase increase. Left, bar graph showing the percentage of cells in each phase of the cell cycle (G0/G1, S, and G2). Right, histogram of flow cytometry overlapping with or without cisplatin treatment. **(E)** XTT assay of three cell lines after 48 h cisplatin (1 µM) treatment. **(F)** XTT assay of U251 wild-type cells treated for 48 h with cisplatin (1 μM, alone), BI-D1870 (1 μM, alone), or the combination of cisplatin plus BI-D1870. **(G)** Representative western blot analysis of three cell lines after cisplatin treatment for 24 h. All bar graphs represent average values of three experiments, with error bars indicating the standard deviation. *, *p* < 0.05; n. s, not significant.

To validate that pathway results from the microarray data were associated with related cellular functions in tumor growth and drug response, we performed *in vitro* experiments. First, to test cell viability in RSK1 KO and RSK2 KO cells, a cell viability (XTT) assay was done. No differences were detected in this assay among the NT, RSK1 KO, and RSK2 KO cell lines at the tested times (Figure 6A). Since the microarray analysis showed that RSK1 KO cells were enriched in DNA replication and cell cycle pathways under 0.1% FBS (unstimulated) conditions, we ran a cell cycle analysis under these same conditions. We found that the RSK1 KO cells were modestly S-phase arrested compared to the NT and RSK2 KO cells; this indicated that RSK1 might regulate cell cycle pathways (Figure 6B) as indicated by the microarray results. We further tested the protein expression level of CDK2, which regulates the cell cycle in S phase (van den Heuvel and Harlow, 1993), and was also identified in the microarray analysis. A Western blot analysis demonstrated that CDK2 was downregulated in RSK1 KO cells under 0.1% FBS conditions (Figure 6C). Interestingly, the CDK2 level was restored after EGF stimulation, which might explain why no major changes were observed in cell viability or number (Figure 6A). To investigate other enriched pathways, the DNA replication/repair pathway in RSK1 KO cells and the immune response pathway in RSK2 KO cells were tested for drug sensitivity by cell cycle analysis and XTT assay. Cisplatin was used as a drug treatment to induce both the DNA damage (Zamble et al., 1996; Reardon et al., 1999) and immune response pathways (Spanos et al., 2009; Hato et al., 2014). The U251 NT cells were cisplatin-resistant, showing modest changes in the cell cycle and no changes in cell viability. Interestingly, cisplatin treatment significantly increased the cell cycle S phase in RSK1 KO cells and resulted in 38% inhibition of cell viability. The RSK2 KO cells also showed S phase arrest in the cell cycle with cisplatin treatment, and up to 36% inhibition of cell viability (Figures 6D,E). The RSK inhibitor BI-D1870, which targets the NTKD of all isoforms, was used in combination with cisplatin in the U251 parental cell line. The drug response increased when cisplatin was used with BI-D1870, compared with cisplatin alone, thereby confirming that RSK activity is important in cisplatin resistance in U251 cells (Figure 6F). We extended our analysis to the DNA replication/repair protein p53 and immune response protein ISG15. The protein expression of p-p53 was increased in both the NT and RSK2 KO cells upon cisplatin treatment, but no induction was recorded in the RSK1 KO cells; this therefore indicated that the DNA replication/repair system was impaired in the RSK1 KO cell line. In addition, ISG15, one of the genes selected from microarray analysis in RSK2 KO cells, showed a marked increase in RSK2 KO cells even before cisplatin treatment, indicating that immune response signaling was elevated in the RSK2 KO cells (Figure 6G).

### differentially expressed genes related to RSK1 and RSK2 are highly correlated *in vitro* and *in vivo*

**FIGURE 7:**
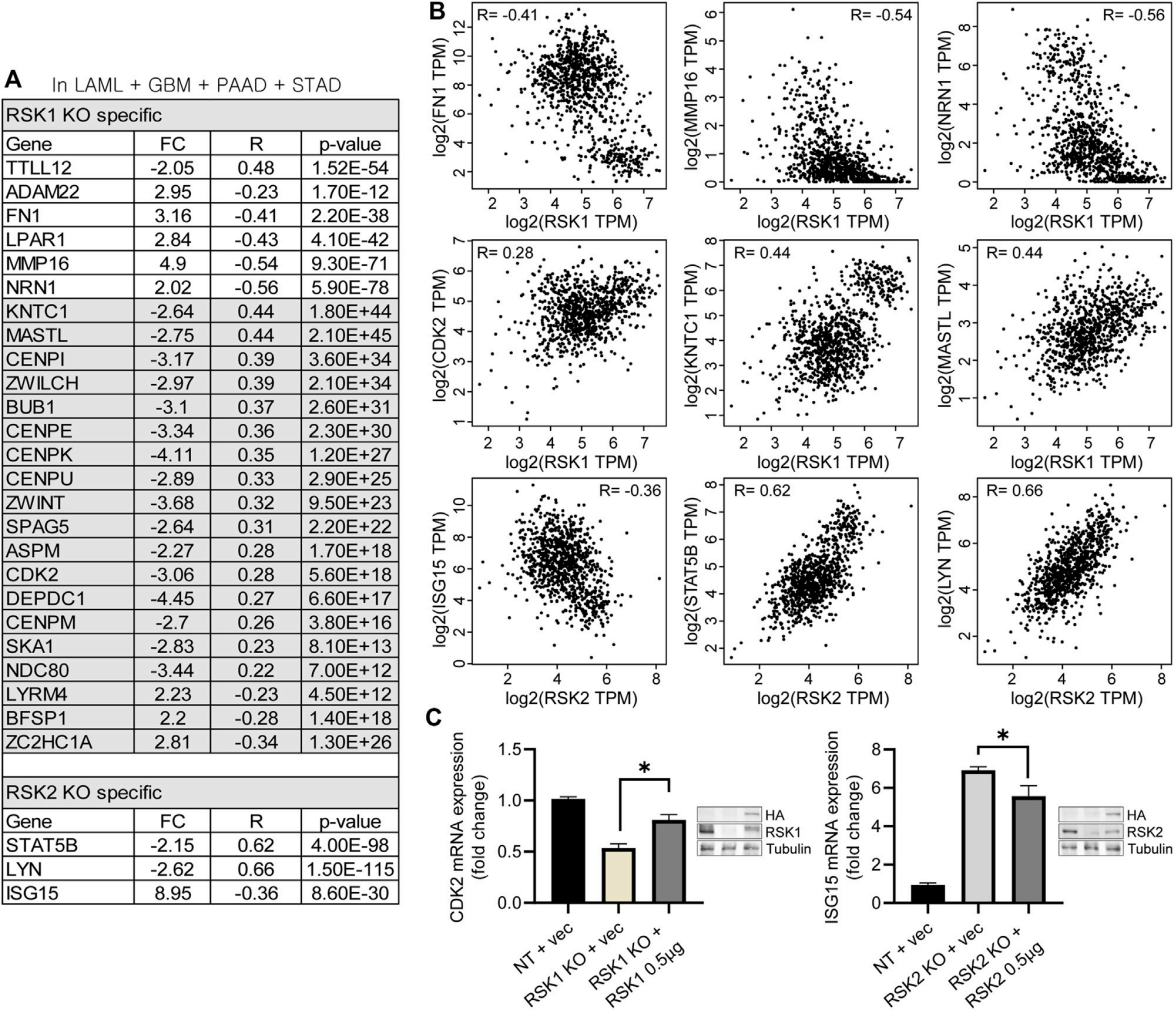
Correlation analysis of DEGs with RSK1 and RSK2. **(A)** Using the TCGA RNA sequencing expression data portal GEPIA2, listed DEGs were highly correlated with RSK1 or RSK2 expression from the TCGA data set. TCGA data sets were from Glioblastoma (GBM), Acute Myeloid Leukemia (LAML), Pancreatic adenocarcinoma (PAAD) and Stomach adenocarcinoma (STAD) patients. Genes were shown with FC (fold changed), R value and *p* values. **(B)** RSK1 was negatively correlated with FN1, MM16 and NRN1, positively correlated with CDK2, KNTC1 and MASTL. RSK2 was negatively correlated with ISG15, positively correlated with STAT5B, and LYN. R: Spearman correlation coefficient. **(C)** CDK2 mRNA expression in RSK1 KO cells with restored RSK1 (left) and ISG15 mRNA expression in RSK2 KO cells with restored RSK2 (right). 0.5 µg of RSK1 and RSK2 were transiently transfected for the assay. The expression of RSK1 and RSK2 protein was confirmed by western blot analysis. * = *p* < 0.05.

To overcome the limitations of microarray results analyzed from a patient-derived cell line, a correlation analysis between the RSK1/2 isoforms and top network related DEGs was conducted using a tumor RNA data set. We conducted a Spearman correlation analysis from the TCGA RNA sequencing expression data portal GEPIA2 (http://gepia2.cancer-pku.cn) (Tang et al., 2017). We used four tumor data sets (LAML, GBM, PAAD, and STAD), all of which showed higher expression of RSK1/2 than non-cancerous tissue (Figure 1C). From twenty genes of “Cellular Development, Cellular Movement, Skeletal and Muscular Disorders” (Figure 3C), six DEGs in the top network related to RSK1 KO (TTLL12, ADMA22, FN1, LPAR1, MMP16, and NRN1) were highly correlated with RSK1 expression, with significant R and *p* values. In addition, from thirty genes of “Cell Cycle, Cellular Assembly and Organization, DNA Replication, Recombination and Repair” in unstimulated conditions, nineteen DEGs were highly correlated with RSK1 expression (ASPM, BFSP1, BUB1, CDK2, CENPE, CENPI, CENPK, CENPM, CENPU, DEPDC1, KNTC1, LYRM4, MASTL, NDC80, SKA1, SPAG5, ZC2HC1A, ZWILCH, and ZWINT). And sixteen genes of ‘Antimicrobial Response, Infectious Diseases and Inflammatory Response’ as the top network of RSK2 KO-specific DEGs were shown to have three DEGs highly correlated with RSK2 expression with significant R and *p* values (ISG15, LYN and STAT5B) (Figure 7A). Each of the three genes from RSK1 and RSK2 network pathways were shown as dot plots that had significant R values in the Spearman correlation analysis (RSK1: FN1, R = −0.41; MMP16, R = −0.54; NRN1, R = −0.56; CDK2, R = 0.28; KNTC1, R = 0.44; and MASL, R = 0.44, RSK2: ISG15, R = −0.36; STAT5B, R = 0.62; and LYN, R = 0.66) (Figure 7B). These results indicate that the DEGs identified in the microarray mRNA analysis are also highly correlated with RSK1 and RSK2 expression in patient tumors. To confirm the DEGs were not the result of off target effects in CRISPR system or the specific cell clone, RSK1 and RSK2 rescue experiments were done in the relevant knockout cell lines. Restoring RSK1 by exogenous expression led to recovery of (from 53% to 81%) CDK2 expression in RSK1 KO cells. Further, restoring RSK2 restored lower levels of (from 6.9 fold to 5.5 fold) ISG15 expression in RSK2 KO cells. This indicates that the changes observed in the knockout cell lines are due to changes in expression of each RSK isoform (Figure 7C).

## Discussion

RSK isoforms are activated by RAS/MAPK pathways and have various biological functions in cancer. In this study, we performed an unbiased, microarray analysis of RSK1 and RSK2 isoform specific gene regulation using CRISPR/cas9 mutated U251 cell lines. The differential expression and various biological functions of RSK isoforms in cancer support the need for development of isoform specific inhibitors. Therefore, we have hypothesized that although RSK family proteins have a high degree of sequence homology, each individual RSK isoform may have both overlapping and specific biological functions in transcriptional regulation. Understanding these differences will be crucial to development of RSKs as targets of new therapeutics.

The pathway analysis of RSK1 KO specific DEGs and cellular assays showed that cell adhesion is modulated byRSK1. RSK were known to regulate cell adhesion *via* modulating several proteins. RSK binds and phosphorylates p120 catenin at serine 320 modulating its proximity cellular partners and re-localization to P-bodies resulting in decreased cell-cell adhesion in melanoma cells (Meant et al., 2020). In addition, our previous study shows that RSK may have a potential role in regulating cell adhesion by phosphorylating Filamin A which is involved in stabilization of the actin cortex (Gawecka et al., 2012).

RSK1 and RSK2 have a direct role in the regulation of cell cycle *via* phosphorylation of several cell cycle checkpoint proteins. Phosphorylation of cyclin dependent kinase (CDK) inhibitor p27 Kip1 on Thr198 results in cytosolic localization promoting G1 phase progression (Fujita et al., 2003; Larrea et al., 2009). RSK has also been shown to promote G2/M transition through phosphorylating Cdc25A and Cdc25B, phosphatases involved in activation of CDK1 (Wu et al., 2014). And RSK2 phosphorylates c-Fos on Ser362 resulting in activation of cyclin D1, promoting G1-S phase progression (David et al., 2005). Consistent with these findings, we found that RSK regulates cell cycle progression also at the transcriptional level. Only RSK1 knockout cells show reduced gene expression related to cell cycle and DNA replication from gene ontology analysis. The pathway results were consistent with those of the KEGG analysis and IPA network pathway analysis and cell cycle assays indicating RSK1 has a specific role in regulating these pathways. We have observed decreased CDK2 protein expression in serum reduced conditions as expected however we found CDK2 expression was recovered when cells were EGF stimulated suggesting that other signaling pathways might recover CDK2 expression. This also explains the lack of effects on proliferation under high FBS conditions.

Interferon-stimulated gene 15 (ISG15) is a member of the ubiquitin-like protein superfamily and induced by type-I IFN (alpha/beta) (Kumar et al., 1993) as well as TNF alpha (Lertsooksawat et al., 2019). ISG15 is conjugated to target proteins by a process called ISGylation to regulate activity of target proteins (Loeb and Haas, 1992). Expression of ISG15 was linked to resistance to cisplatin treatment in tumor cells, suggesting ISG15 is a biomarker for drug sensitivity (Huo et al., 2017; Wang et al., 2020) which is consistent with our data showing increased ISG15 and induced cisplatin sensitivity in RSK2 knockout cells.

We used IPA to identify potential upstream regulators including transcription factors connected with the differentially expressed genes (DEGs) (Kramer et al., 2014). Upstream regulator analysis identified 38 potential regulators of the transcription of multiple genes involved in cell cycle and DNA replication from RSK1 knockout DEGs and 23 potential regulators involved in immune response from RSK2 knockout DEGs. Further studies are required to determine if these proteins are regulated by RSK1 or RSK2 isoforms specifically. Me´ant *et al* identified RSK isoform specific binding partners in HEK293 cells by using proximity-dependent biotinylation (BioID) approaches (Meant et al., 2020). DNA replication proteins PCNA (Dianova et al., 2001), ORC5 (Springer et al., 1999) and CDC45 (Liu et al., 2006) were identified as RSK1 specific binding proteins. No proteins were identified as RSK2 binding proteins related to immune response.

In conclusion, our study identifies isoform specific transcriptional programs for RSK1 and RSK2. Current RSK inhibitors affect all isoforms and these are being pursued as potential therapeutic agents for diverse cancers including GBM, melanoma, prostate, and breast cancers. This analysis suggests that isoform specific inhibitors may be needed to more precisely target RSK functions and thereby improve therapeutic efficacy and reduce complications that may result from hitting multiple RSK isoforms.

## Data Availability

The data presented in the study are deposited in the Gene Expression Omnibus (GEO) repository, accession number GSE213592.

## References

[B1] Anjum R. Blenis J. (2008). The RSK family of kinases: Emerging roles in cellular signalling. Nat. Rev. Mol. Cell Biol. 9, 747–758. 18813292 10.1038/nrm2509

[B2] Bignone P. A. Lee K. Y. Liu Y. Emilion G. Finch J. Soosay A. E. (2007). RPS6KA2, a putative tumour suppressor gene at 6q27 in sporadic epithelial ovarian cancer. Oncogene 26, 683–700. 16878154 10.1038/sj.onc.1209827

[B3] Bradner J. E. Hnisz D. Young R. A. (2017). Transcriptional addiction in cancer. Cell 168, 629–643. 10.1016/j.cell.2016.12.013 28187285 PMC5308559

[B4] Bruning J. C. Gillette J. A. Zhao Y. Bjorbaeck C. Kotzka J. Knebel B. (2000). Ribosomal subunit kinase-2 is required for growth factor-stimulated transcription of the c-Fos gene. Proc. Natl. Acad. Sci. U. S. A. 97, 2462–2467. 10.1073/pnas.97.6.2462 10716983 PMC15951

[B5] Chen R. H. Abate C. Blenis J. (1993). Phosphorylation of the c-Fos transrepression domain by mitogen-activated protein kinase and 90-kDa ribosomal S6 kinase. Proc. Natl. Acad. Sci. U. S. A. 90, 10952–10956. 10.1073/pnas.90.23.10952 8248197 PMC47899

[B6] Chen R. H. Juo P. C. Curran T. Blenis J. (1996). Phosphorylation of c-Fos at the C-terminus enhances its transforming activity. Oncogene 12, 1493–1502. 8622865

[B7] David J. P. Mehic D. Bakiri L. Schilling A. F. Mandic V. Priemel M. (2005). Essential role of RSK2 in c-Fos-dependent osteosarcoma development. J. Clin. Invest 115, 664–672. 10.1172/JCI22877 15719069 PMC548699

[B8] Davis I. J. Hazel T. G. Chen R. H. Blenis J. Lau L. F. (1993). Functional domains and phosphorylation of the orphan receptor Nur77. Mol. Endocrinol. 7, 953–964. 10.1210/mend.7.8.8232315 8232315

[B9] De Cesare D. Jacquot S. Hanauer A. Sassone-Corsi P. (1998). Rsk-2 activity is necessary for epidermal growth factor-induced phosphorylation of CREB protein and transcription of c-fos gene. Proc. Natl. Acad. Sci. U. S. A. 95, 12202–12207. 10.1073/pnas.95.21.12202 9770464 PMC22809

[B10] Dianova, II Bohr V. A. Dianov G. L. (2001). Interaction of human AP endonuclease 1 with flap endonuclease 1 and proliferating cell nuclear antigen involved in long-patch base excision repair. Biochemistry 40, 12639–12644. 10.1021/bi011117i 11601988

[B11] Doehn U. Hauge C. Frank S. R. Jensen C. J. Duda K. Nielsen J. V. (2009). RSK is a principal effector of the RAS-ERK pathway for eliciting a coordinate promotile/invasive gene program and phenotype in epithelial cells. Mol. Cell 35, 511–522. 10.1016/j.molcel.2009.08.002 19716794 PMC3784321

[B12] Dummler B. A. Hauge C. Silber J. Yntema H. G. Kruse L. S. Kofoed B. (2005). Functional characterization of human RSK4, a new 90-kDa ribosomal S6 kinase, reveals constitutive activation in most cell types. J. Biol. Chem. 280, 13304–13314. 10.1074/jbc.M408194200 15632195

[B13] Erazo T. Moreno A. Ruiz-Babot G. Rodriguez-Asiain A. Morrice N. A. Espadamala J. (2013). Canonical and kinase activity-independent mechanisms for extracellular signal-regulated kinase 5 (ERK5) nuclear translocation require dissociation of Hsp90 from the ERK5-Cdc37 complex. Mol. Cell Biol. 33, 1671–1686. 10.1128/MCB.01246-12 23428871 PMC3624243

[B14] Fujita N. Sato S. Tsuruo T. (2003). Phosphorylation of p27Kip1 at threonine 198 by p90 ribosomal protein S6 kinases promotes its binding to 14-3-3 and cytoplasmic localization. J. Biol. Chem. 278, 49254–49260. 10.1074/jbc.M306614200 14504289

[B15] Gawecka J. E. Young-Robbins S. S. Sulzmaier F. J. Caliva M. J. Heikkila M. M. Matter M. L. (2012). RSK2 protein suppresses integrin activation and fibronectin matrix assembly and promotes cell migration. J. Biol. Chem. 287, 43424–43437. 10.1074/jbc.M112.423046 23118220 PMC3527930

[B16] Ghoda L. Lin X. Greene W. C. (1997). The 90-kDa ribosomal S6 kinase (pp90rsk) phosphorylates the N-terminal regulatory domain of IkappaBalpha and stimulates its degradation *in vitro* . J. Biol. Chem. 272, 21281–21288. 10.1074/jbc.272.34.21281 9261139

[B17] Hato S. V. Khong A. DE Vries I. J. Lesterhuis W. J. (2014). Molecular pathways: The immunogenic effects of platinum-based chemotherapeutics. Clin. Cancer Res. 20, 2831–2837. 10.1158/1078-0432.CCR-13-3141 24879823

[B18] Hazzalin C. A. Mahadevan L. C. (2002). MAPK-Regulated transcription: A continuously variable gene switch? Nat. Rev. Mol. Cell Biol. 3, 30–40. 10.1038/nrm715 11823796

[B19] Hill C. S. Treisman R. (1995). Transcriptional regulation by extracellular signals: Mechanisms and specificity. Cell 80, 199–211. 10.1016/0092-8674(95)90403-4 7834740

[B20] Huang Da W. Sherman B. T. Lempicki R. A. (2009a). Bioinformatics enrichment tools: Paths toward the comprehensive functional analysis of large gene lists. Nucleic Acids Res. 37, 1–13. 10.1093/nar/gkn923 19033363 PMC2615629

[B21] Huang Da W. Sherman B. T. Lempicki R. A. (2009b). Systematic and integrative analysis of large gene lists using DAVID bioinformatics resources. Nat. Protoc. 4, 44–57. 10.1038/nprot.2008.211 19131956

[B22] Huo Y. Zong Z. Wang Q. Zhang Z. Deng H. (2017). ISG15 silencing increases cisplatin resistance via activating p53-mediated cell DNA repair. Oncotarget 8, 107452–107461. 10.18632/oncotarget.22488 29296177 PMC5746079

[B23] Joel P. B. Smith J. Sturgill T. W. Fisher T. L. Blenis J. Lannigan D. A. (1998). pp90rsk1 regulates estrogen receptor-mediated transcription through phosphorylation of Ser-167. Mol. Cell Biol. 18, 1978–1984. 10.1128/mcb.18.4.1978 9528769 PMC121427

[B24] Kang S. Elf S. Lythgoe K. Hitosugi T. Taunton J. Zhou W. (2010). p90 ribosomal S6 kinase 2 promotes invasion and metastasis of human head and neck squamous cell carcinoma cells. J. Clin. Invest 120, 1165–1177. 10.1172/JCI40582 20234090 PMC2846050

[B25] Kohn M. Hameister H. Vogel M. Kehrer-Sawatzki H. (2003). Expression pattern of the Rsk2, Rsk4 and Pdk1 genes during murine embryogenesis. Gene Expr. Patterns 3, 173–177. 10.1016/s1567-133x(03)00004-8 12711546

[B26] Kramer A. Green J. Pollard J. Tugendreich S. (2014). Causal analysis approaches in ingenuity pathway analysis. Bioinformatics 30, 523–530. 10.1093/bioinformatics/btt703 24336805 PMC3928520

[B27] Kumar S. Yoshida Y. Noda M. (1993). Cloning of a cDNA which encodes a novel ubiquitin-like protein. Biochem. Biophys. Res. Commun. 195, 393–399. 10.1006/bbrc.1993.2056 8395831

[B28] Lara R. Mauri F. A. Taylor H. Derua R. Shia A. Gray C. (2011). An siRNA screen identifies RSK1 as a key modulator of lung cancer metastasis. Oncogene 30, 3513–3521. 10.1038/onc.2011.61 21423205

[B29] Larrea M. D. Hong F. Wander S. A. Da Silva T. G. Helfman D. Lannigan D. (2009). RSK1 drives p27Kip1 phosphorylation at T198 to promote RhoA inhibition and increase cell motility. Proc. Natl. Acad. Sci. U. S. A. 106, 9268–9273. 10.1073/pnas.0805057106 19470470 PMC2695095

[B30] Lee T. I. Young R. A. (2013). Transcriptional regulation and its misregulation in disease. Cell 152, 1237–1251. 10.1016/j.cell.2013.02.014 23498934 PMC3640494

[B31] Lertsooksawat W. Wongnoppavich A. Chairatvit K. (2019). Up-regulation of interferon-stimulated gene 15 and its conjugation machinery, UbE1L and UbcH8 expression by tumor necrosis factor-alpha through p38 MAPK and JNK signaling pathways in human lung carcinoma. Mol. Cell Biochem. 462, 51–59. 10.1007/s11010-019-03609-5 31428903

[B32] Liu P. Barkley L. R. Day T. Bi X. Slater D. M. Alexandrow M. G. (2006). The Chk1-mediated S-phase checkpoint targets initiation factor Cdc45 via a Cdc25A/Cdk2-independent mechanism. J. Biol. Chem. 281, 30631–30644. 10.1074/jbc.M602982200 16912045

[B33] Loeb K. R. Haas A. L. (1992). The interferon-inducible 15-kDa ubiquitin homolog conjugates to intracellular proteins. J. Biol. Chem. 267, 7806–7813. 10.1016/s0021-9258(18)42585-9 1373138

[B34] Malireddi R. K. S. Gurung P. Kesavardhana S. Samir P. Burton A. Mummareddy H. (2020). Innate immune priming in the absence of TAK1 drives RIPK1 kinase activity-independent pyroptosis, apoptosis, necroptosis, and inflammatory disease. J. Exp. Med. 217, 20191644. 10.1084/jem.20191644 PMC7062518 31869420

[B35] Meant A. Gao B. Lavoie G. Nourreddine S. Jung F. Aubert L. (2020). Proteomic analysis reveals a role for RSK in p120-catenin phosphorylation and melanoma cell-cell adhesion. Mol. Cell Proteomics 19, 50–64. 10.1074/mcp.RA119.001811 31678930 PMC6944238

[B36] Pankov R. Yamada K. M. (2002). Fibronectin at a glance. J. Cell Sci. 115, 3861–3863. 10.1242/jcs.00059 12244123

[B37] Reardon J. T. Vaisman A. Chaney S. G. Sancar A. SancAr A. (1999). Efficient nucleotide excision repair of cisplatin, oxaliplatin, and Bis-aceto-ammine-dichloro-cyclohexylamine-platinum(IV) (JM216) platinum intrastrand DNA diadducts. Cancer Res. 59, 3968–3971. 10463593

[B38] Rivera V. M. Miranti C. K. Misra R. P. Ginty D. D. Chen R. H. Blenis J. (1993). A growth factor-induced kinase phosphorylates the serum response factor at a site that regulates its DNA-binding activity. Mol. Cell Biol. 13, 6260–6273. 10.1128/mcb.13.10.6260 8413226 PMC364685

[B39] Romeo Y. Roux P. P. (2011). Paving the way for targeting RSK in cancer. Expert Opin. Ther. Targets 15, 5–9. 10.1517/14728222.2010.531014 20958120

[B40] Romeo Y. Zhang X. Roux P. P. (2012). Regulation and function of the RSK family of protein kinases. Biochem. J. 441, 553–569. 10.1042/BJ20110289 22187936

[B41] Sanjana N. E. Shalem O. Zhang F. (2014). Improved vectors and genome-wide libraries for CRISPR screening. Nat. Methods 11, 783–784. 10.1038/nmeth.3047 25075903 PMC4486245

[B42] Schouten G. J. Vertegaal A. C. Whiteside S. T. Israel A. Toebes M. Dorsman J. C. (1997). IkappaB alpha is a target for the mitogen-activated 90 kDa ribosomal S6 kinase. EMBO J. 16, 3133–3144. 10.1093/emboj/16.11.3133 9214631 PMC1169932

[B43] Shi G. X. Yang W. S. Jin L. Matter M. L. Ramos J. W. (2018). RSK2 drives cell motility by serine phosphorylation of LARG and activation of Rho GTPases. Proc. Natl. Acad. Sci. U. S. A. 115, E190–E199. 10.1073/pnas.1708584115 29279389 PMC5777029

[B44] Shimamura A. Ballif B. A. Richards S. A. Blenis J. (2000). Rsk1 mediates a MEK-MAP kinase cell survival signal. Curr. Biol. 10, 127–135. 10.1016/s0960-9822(00)00310-9 10679322

[B45] Spanos W. C. Nowicki P. Lee D. W. Hoover A. Hostager B. Gupta A. (2009). Immune response during therapy with cisplatin or radiation for human papillomavirus-related head and neck cancer. Arch. Otolaryngol. Head. Neck Surg. 135, 1137–1146. 10.1001/archoto.2009.159 19917928

[B46] Springer J. Kneissl M. Putter V. Grummt F. (1999). Identification and characterization of MmORC4 and MmORC5, two subunits of the mouse origin of replication recognition complex. Chromosoma 108, 243–249. 10.1007/s004120050374 10460412

[B47] Sulzmaier F. J. Ramos J. W. (2013). RSK isoforms in cancer cell invasion and metastasis. Cancer Res. 73, 6099–6105. 10.1158/0008-5472.CAN-13-1087 24097826 PMC3801100

[B48] Sulzmaier F. J. Young-Robbins S. Jiang P. Geerts D. Prechtl A. M. Matter M. L. (2016). RSK2 activity mediates glioblastoma invasiveness and is a potential target for new therapeutics. Oncotarget 7, 79869–79884. 10.18632/oncotarget.13084 27829215 PMC5346757

[B49] Tang Z. Li C. Kang B. Gao G. Li C. Zhang Z. (2017). Gepia: A web server for cancer and normal gene expression profiling and interactive analyses. Nucleic Acids Res. 45, W98–W102. 10.1093/nar/gkx247 28407145 PMC5570223

[B50] Thakur A. Sun Y. Bollig A. Wu J. Biliran H. Banerjee S. (2008). Anti-invasive and antimetastatic activities of ribosomal protein S6 kinase 4 in breast cancer cells. Clin. Cancer Res. 14, 4427–4436. 10.1158/1078-0432.CCR-08-0458 18628456 PMC3771666

[B51] To W. S. Midwood K. S. (2011). Plasma and cellular fibronectin: Distinct and independent functions during tissue repair. Fibrogenes. Tissue Repair 4, 21. 10.1186/1755-1536-4-21 PMC3182887 21923916

[B52] Van Den Heuvel S. Harlow E. (1993). Distinct roles for cyclin-dependent kinases in cell cycle control. Science 262, 2050–2054. 10.1126/science.8266103 8266103

[B53] Wang J. M. Liu B. Q. Zhang Q. Hao L. Li C. Yan J. (2020). ISG15 suppresses translation of ABCC2 via ISGylation of hnRNPA2B1 and enhances drug sensitivity in cisplatin resistant ovarian cancer cells. Biochim. Biophys. Acta Mol. Cell Res. 1867, 118647. 10.1016/j.bbamcr.2020.118647 31926942

[B54] Whitmarsh A. J. Davis R. J. (2000). Regulation of transcription factor function by phosphorylation. Cell Mol. Life Sci. 57, 1172–1183. 10.1007/pl00000757 11028910 PMC11146857

[B55] Whitmarsh A. J. (2007). Regulation of gene transcription by mitogen-activated protein kinase signaling pathways. Biochim. Biophys. Acta 1773, 1285–1298. 10.1016/j.bbamcr.2006.11.011 17196680

[B56] Wingate A. D. Campbell D. G. Peggie M. Arthur J. S. (2006). Nur77 is phosphorylated in cells by RSK in response to mitogenic stimulation. Biochem. J. 393, 715–724. 10.1042/BJ20050967 16223362 PMC1360724

[B57] Wu C. F. Liu S. Lee Y. C. Wang R. Sun S. Yin F. (2014). RSK promotes G2/M transition through activating phosphorylation of Cdc25A and Cdc25B. Oncogene 33, 2385–2394. 10.1038/onc.2013.182 23708659 PMC4026278

[B58] Wu C. Orozco C. Boyer J. Leglise M. Goodale J. Batalov S. (2009). BioGPS: An extensible and customizable portal for querying and organizing gene annotation resources. Genome Biol. 10, R130. 10.1186/gb-2009-10-11-r130 19919682 PMC3091323

[B59] Yamnik R. L. Holz M. K. (2010). mTOR/S6K1 and MAPK/RSK signaling pathways coordinately regulate estrogen receptor alpha serine 167 phosphorylation. FEBS Lett. 584, 124–128. 10.1016/j.febslet.2009.11.041 19925796 PMC8117181

[B60] Yang S. H. Sharrocks A. D. Whitmarsh A. J. (2003). Transcriptional regulation by the MAP kinase signaling cascades. Gene 320, 3–21. 10.1016/s0378-1119(03)00816-3 14597384

[B61] Yang W. Ju J. H. Lee K. M. Nam K. Oh S. Shin I. (2013). Protein kinase B/Akt1 inhibits autophagy by down-regulating UVRAG expression. Exp. Cell Res. 319, 122–133. 10.1016/j.yexcr.2012.11.014 23200933

[B62] Yang X. Matsuda K. Bialek P. Jacquot S. Masuoka H. C. Schinke T. (2004). ATF4 is a substrate of RSK2 and an essential regulator of osteoblast biology; implication for Coffin-Lowry Syndrome. Cell 117, 387–398. 10.1016/s0092-8674(04)00344-7 15109498

[B63] Zamble D. B. Mu D. Reardon J. T. Sancar A. Lippard S. J. (1996). Repair of cisplatin-DNA adducts by the mammalian excision nuclease. Biochemistry 35, 10004–10013. 10.1021/bi960453 8756462

